# Prevalence of myopia among children and adolescents aged 6–16 during COVID-19 pandemic: a large-scale cross-sectional study in Tianjin, China

**DOI:** 10.1136/bjo-2023-323688

**Published:** 2023-07-06

**Authors:** Tongtong Li, Ruihua Wei, Bei Du, Qi Wu, Jing Yan, Xiangda Meng, Yuanyuan Liu, Qiang Yang, Chea-Su Kee, Guowei Huang, Hua Yan

**Affiliations:** 1 School of Public Health, Tianjin Medical University, Tianjin, China; 2 Tianjin Key Laboratory of Ocular Trauma, Tianjin, China; 3 Tianjin Key Laboratory of Retinal Functions and Diseases, Tianjin Branch of National Clinical Research Center for Ocular Disease, Eye Institute and School of Optometry, Tianjin Medical University Eye Hospital, Tianjin, China; 4 Department of Ophthalmology, Tianjin Medical University General Hospital, Laboratory of Molecular Ophthalmology, Tianjin Medical University, Tianjin, China; 5 Shenyang Xingqi Pharmaceutical Company, Ltd, Shenyang, China; 6 School of Optometry, Centre for Myopia Research, Research Centre for SHARP Vision, The Hong Kong Polytechnic University, Hong Kong, China; 7 School of Medicine, Nankai University, Tianjin, China

**Keywords:** Vision

## Abstract

**Purpose:**

This study aimed to determine the prevalence of myopia among children and adolescents aged 6–16 years during COVID-19 pandemic in Tianjin, China.

**Methods:**

This was a cross-sectional study using data from the Tianjin Child and Adolescent Research of Eye between March and June in 2021. A total of 909 835 children and adolescents aged 6–16 years from 1348 primary and secondary schools in Tianjin, China were recruited. Prevalence of myopia with 95% CIs was described in different regions, sexes and ages. The regions-standardised prevalence rate and chain growth rate of myopia in different ages were described the characteristics of myopia.

**Results:**

A total of 864 828 participants (95.05% participation rate) were included in the analysis. The age range was 6–16 with a mean age of 11.50±2.79 years. The overall prevalence of myopia was 54.71% (95% CI 54.60% to 54.81%). The prevalence of myopia was 57.58% (95% CI 57.43% to 57.73%) for girls and 52.05% (95% CI 51.91% to 52.20%) for boys. Students living in the six central districts had the highest prevalence of moderate myopia (19.09% (95% CI 19.01% to 19.17%)) and high myopia (5.43% (95% CI 5.39% to 5.48%)). The regions-standardised prevalence of myopia was increased by age and the highest chain growth rate of myopia was up to 47.99% at 8 years.

**Conclusions:**

The prevalence of myopia in Tianjin is high during COVID-19 pandemic. The progression of myopia started to increase dramatically at 8 years, and the increasing slowed down at 14 years. For policy-makers, intervention in the lower age groups may be important to control myopia progression.

**WHAT IS ALREADY KNOWN ON THIS TOPIC**

- Although several school-based cross-sectional studies were performed in mainland China, large-scale and city-wide studies in school-aged children during COVID-19 pandemic should be provided.

**WHAT THIS STUDY ADDS**

- This city-wide scale observation revealed the prevalence of myopia during COVID-19 pandemic. The regions-standardised prevalence of myopia was increased by age, and the highest chain growth rate of myopia was 47.99% at 8 years.

**HOW THIS STUDY MIGHT AFFECT RESEARCH, PRACTICE OR POLICY**

- For policy-makers, intervention in young kids may be crucial to control progression of myopia.

## Introduction

Myopia is the most common cause of visual impairment.^1^ Although myopia is a widespread issue, east Asian countries sharing high-pressure educational systems, including China, have seen the fastest growth and highest prevalence.^2 3^ Nationwide data from China in 2018 revealed, for instance, that an estimated 81.0% of students who have completed secondary school were myopic, and 21.9% of senior high school students have high myopia.^4^ According to a study from Weifang, Shandong province, China, the prevalence of myopia was 93.14% among high school students and the prevalence of high myopia was 25.12% among students completing secondary education in 2020.^5^ High myopia significantly raises the risk of glaucoma, macular atrophy, pathological myopia and other serious vision loss conditions.^6 7^

Due to the COVID-19 causing a rare global pandemic, various lockdown measures have been imposed on populations everywhere, including limited outdoor activities and school closures to contain to spread of the virus. Students completed distance education at home at the end of January 2020. It was speculated that school closures and online studies might have a negative impact on children’s physical and mental health.^8^ International and domestic researchers were concerned that significantly decreased time spent outdoors and increased screen time at home may cause an increase in myopia among children and adolescents.^9–11^ Thus, it is urgent to investigate the onset and development of myopia among children and adolescents during learning at home.

Although several cross-sectional school-based studies on myopia were performed in mainland China, while such a large sample size and covering the whole city level during COVID-19 pandemic, there are relatively fewer. Tianjin has six central districts, four districts adjacent to the centre, and five suburbs. Individual districts differ in terms of living habits, educational level, financial condition, and medical and health resources. Therefore, the distribution of myopia among children and adolescents may also be different. The current large-scale cross-sectional study aimed to assess the prevalence of myopia among children and adolescents aged 6–16 during COVID-19 pandemic in Tianjin, China and to provide relevant scientific data for decision-makers.

## Methods

### Study population

**Figure 1:**
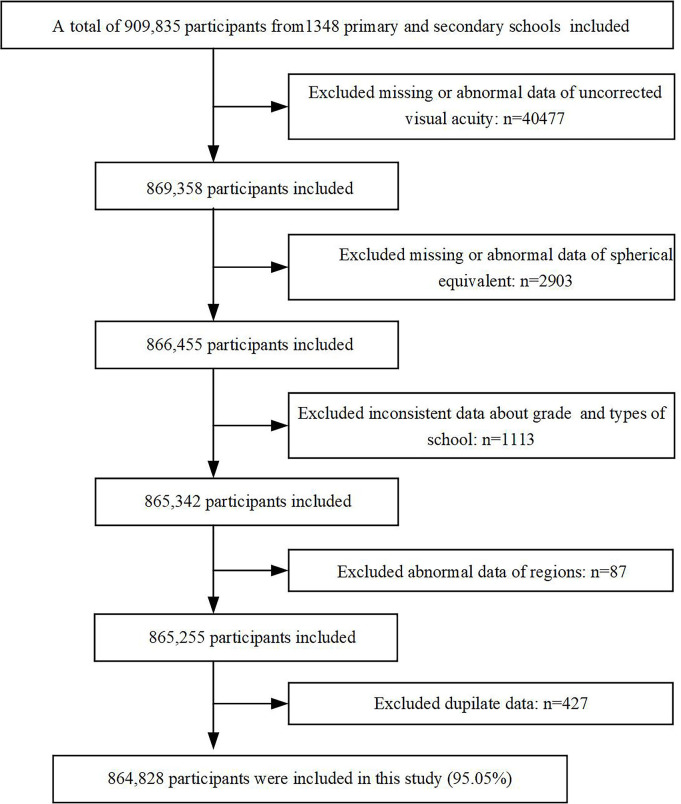
Flow chart of participants recruitment.

This was a cross-sectional study using data from the Tianjin Child and Adolescent Research of Eye (TCARE). This large-scale study was performed on children and adolescents aged 6–16 years in 1348 primary and secondary schools in 15 districts of Tianjin, China, from March to June in 2021. Exclusion criteria: students who have previously undergone cataract surgery, laser refractive surgery or low-dose atropine were not included. A total of 909 835 participants were eligible for this screen and 864 828 participants (95.05% participation rate) completed all examinations. Figure 1 shows a flow chart of participants recruitment.

### Visual acuity test and refraction screening

Students were instructed to remove the spectacle glasses for the refraction and uncorrected visual acuity (UCVA) examinations. The refraction and visual acuity examinations were performed for all subjects’ eyes by professional opticians who had received standardised training. All examined results were uploaded in real time through the mobile APP.

UCVA was tested using a standard logarithmic visual acuity E chart. When the oculus dexter (OD) was tested, a non-contact, black spoon-shaped eye occlude was used to cover the oculus sinister (OS) from a distance of 5 m. After a 5–10 s interval following the completion of the OD test, the OS was tested. Each subject obtained the break so that their OS could recover from the earlier occlusion. Acuity was tested with and without refractive correction for those wearing spectacles. The results were recorded in the column of corrected visual acuity for the participants who worn contact lenses or orthokeratology lenses.

Non-cycloplegic refractive error was tested using the Tianle RM-9600 autorefractor (Shanghai, China). Each subject underwent non-cycloplegia refraction three times in each eye and the average value was adopted. Each subject was reexamined if the differences between any two results of the three results obtained were more than 0.50 dioptres (D). The regular formula of the algebraic sum of the dioptric powers of the sphere and half of the cylinder (sphere+0.5 cylinder) was used to determine the spherical equivalent (SE). Myopia was defined as an SE refraction of <−0.50 D when the UCVA<5.0 and graded into mild myopia (−3.00D≤SE<−0.50D), moderate myopia (−6.00D≤SE<−3.00D) and high myopia (SE<−6.00D).^12^

### Statistical analyses

IBM SPSS V.24.0 was used to analyse all data. Figures were performed using Graphpad Prism V.9.4.1 and Arcgis V.10.8. Normally distributed continuous variables were described by mean and SD and skewed continuous variables were described by medians and IQR. While categorical variables were described by number of cases and percentage. The prevalence of myopia with 95% CIs was described in different regions, sexes and ages. The regions-standardised prevalence rate and chain growth rate of myopia in different ages were described the characteristics of myopia among children and adolescents during COVID-19 pandemic in Tianjin, China. Given the influence of a large sample size, hypothesis testing was not performed.

## Results

A total of 864 828 participants were included in this study. The age range was 6–16 with a mean age of 11.50±2.79 years. Of the participants, 449 414 (51.97%) were boys and 415 414 (48.03%) were girls. The overall prevalence of myopia was 54.71% (95% CI 54.60% to 54.81%). The characteristics of participants with and without myopia were shown in table 1.

**Table 1:** Characteristics of participants with and without myopia

Characteristics	Total	Myopia	
	(n=864 828)	No (n=391 711)	Yes (n=473 117)
Age, mean±SD, years	11.50±2.79	10.16±2.44	12.60±2.56
Sex			
Male, no (%)	449 414 (51.97)	215 479 (47.95)	233 935 (52.05)
Female, no (%)	415 414 (48.03)	176 232 (42.42)	239 182 (57.58)
SE of right eye, M (P_25_, P_75_), D	−0.88 (−2.63, 0.00)	0.00 (−0.38, 0.38)	−2.38 (−3.88, –1.25)
SE of left eye, M (P_25_, P_75_), D	−0.75 (−2.50, 0.00)	0.00 (−0.38, 0.38)	−2.25 (−3.68, –1.00)
UCVA of right eye, mean±SD	4.72±0.37	5.00±0.18	4.50±0.33
UCVA of left eye, mean±SD	4.74±0.36	5.00±0.17	4.53±0.33
Regions			
Six central districts, no (%)	306 172 (35.40)	141 824 (46.32)	164 348 (53.68)
Four districts adjacent to the centre, no (%)	174 632 (20.19)	81 316 (46.56)	93 316 (53.44)
Suburb, no (%)	384 024 (44.40)	168 571 (43.90)	215 453 (56.10)

D, dioptre; SE, spherical equivalent; UCVA, uncorrected visual acuity.

**Figure 2:**
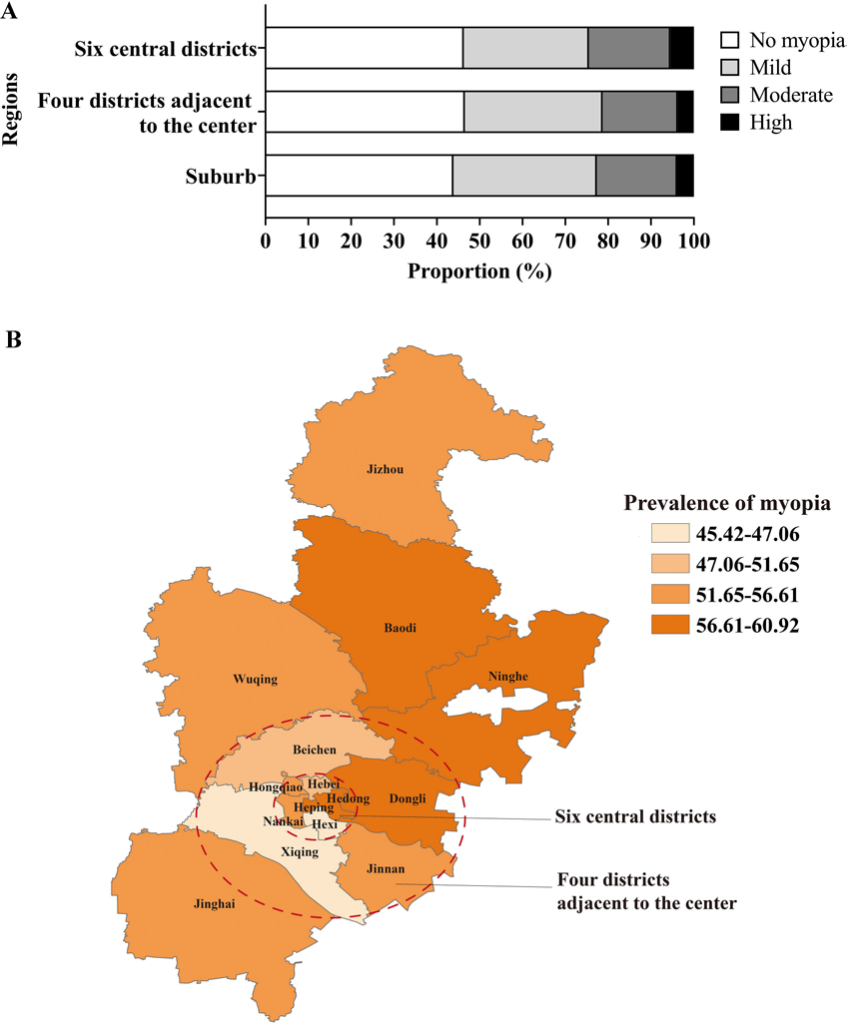
The distribution of severity of myopia in the different regions (A) and the spatial distribution map of the prevalence of myopia (B) in Tianjin, China. Six central districts: Heping, Hebei, Hedong, Hexi, Hongqiao, Nankai; Four districts adjacent to the centre: Beichen, Dongli, Jinnan, Xiqing; Suburb: Baodi, Jinghai, Jizhou, Ninghe, Wuqing.

**Figure 3:**
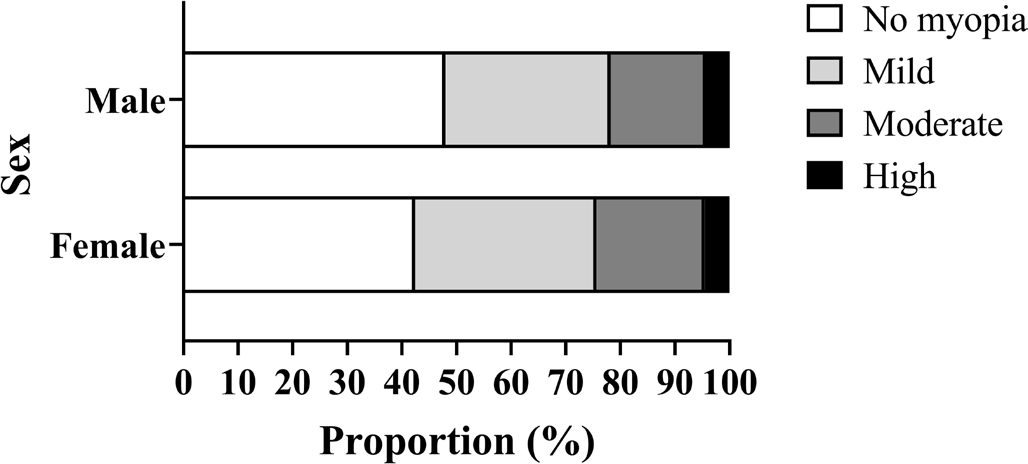
The distribution of severity of myopia in different sexes.

The prevalence of total myopia in six central districts, four districts adjacent to the centre and suburb was 53.68% (95% CI 53.57% to 53.78%), 53.44% (95% CI 53.33% to 53.54%), 56.10% (95% CI 56.00% to 56.21%), respectively. The participants living in the six central districts had the highest prevalence of moderate myopia (19.09% (95% CI 19.01% to 19.17%)) and high myopia (5.43% (95% CI 5.39% to 5.48%)), compared with other regions (figure 2A). The spatial distribution map of the prevalence of myopia in 15 districts of Tianjin was shown in figure 2B. Stratified by sex, the prevalence of total myopia and mild, moderate and high myopia was higher in girls (57.58% (95% CI 57.43% to 57.73%); 33.21% (95% CI 33.07% to 33.36%); 19.82% (95% CI 19.70% to 19.94%); 4.54% (95% CI 4.48% to 4.60%)) than that in boys (52.05% (95% CI 51.91% to 52.20%); 30.24% (95% CI 30.10% to 30.37%); 17.54% (95% CI 17.42% to 17.65%); 4.28% (95% CI 4.22% to 4.34%)), respectively (figure 3).

**Figure 4:**
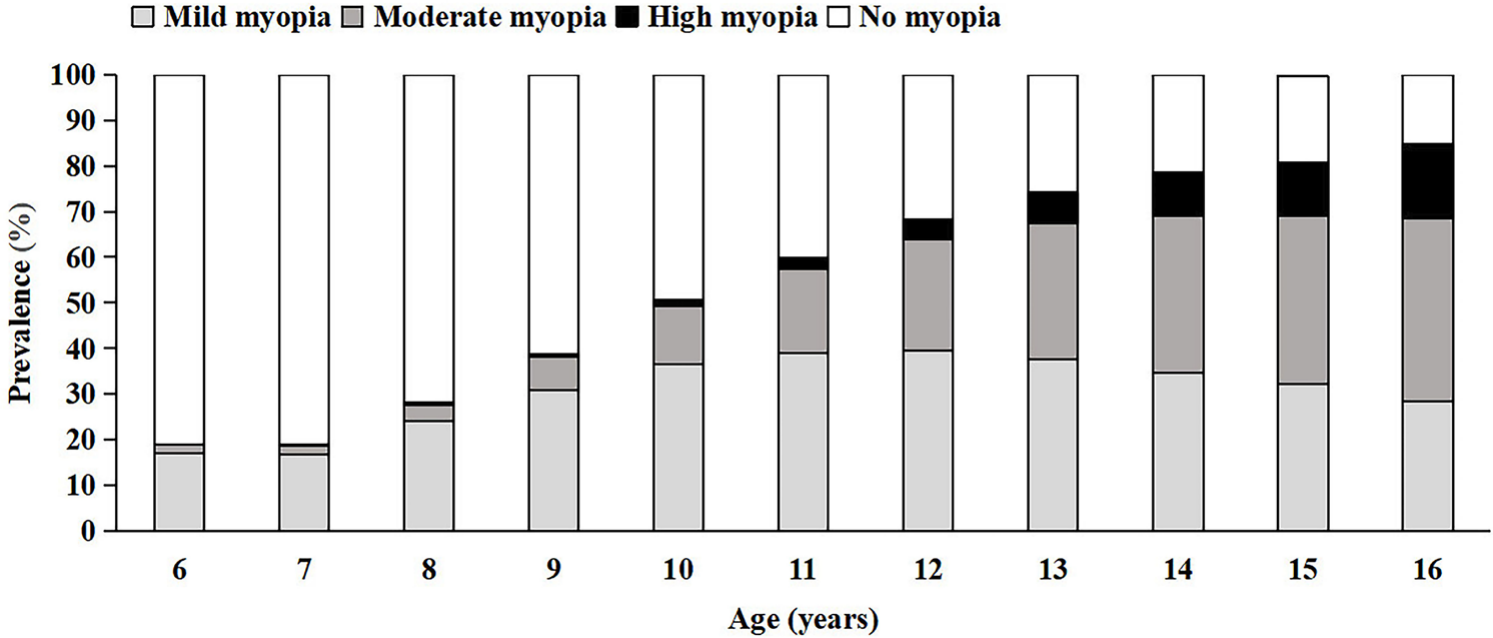
Prevalence of myopia in different ages.

Regarding the age, the prevalence of total myopia increased gradually by age. The change was observed in both moderate and high myopia (figure 4). The prevalence of total myopia was 19.04% (95% CI 18.96% to 19.12%) at 6 years and increased to 84.81% (95% CI 84.74% to 84.89%) at 16 years. The prevalence of high myopia was up to 16.25% (95% CI 15.94% to 16.56%) at 16 years (online supplemental e table 1).

10.1136/bjo-2023-323688.supp1 Supplementary data

**Figure 5:**
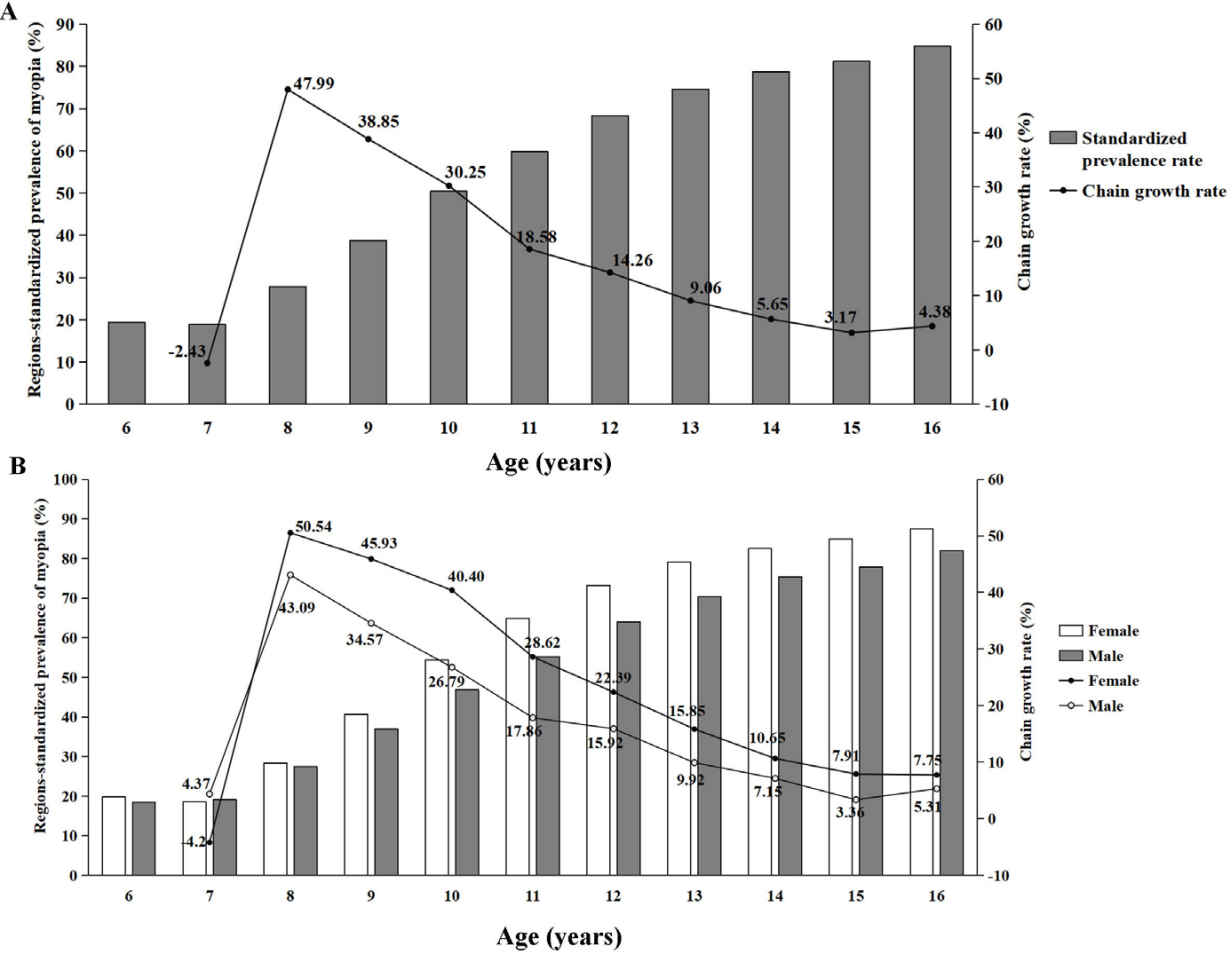
The regions-standardised prevalence and chain growth rate of myopia in different ages (A). The regions-standardised prevalence and chain growth rate of myopia in different ages stratified by sex (B).

With the further analysis of the data, the regions-standardised prevalence of myopia was also increased by age and the highest chain growth rate of myopia was 47.99% at 8 years, with the increasing slow down at 14 years (figure 5A). These findings were also observed stratified by sex (figure 5B).

## Discussion

The school-based, large-scale, city-wide, cross-sectional study evaluated the prevalence and distribution of myopia among children and adolescents aged 6–16 years during COVID-19 pandemic in Tianjin. The overall prevalence of myopia was 54.71%. Girls had a higher prevalence of myopia than boys and participants living in the six central districts had the highest prevalence of moderate myopia and high myopia. The progression started to increase dramatically at 8 years, and the increase slowed down at 14 years.

It was reported that the prevalence of myopia among Chinese preschool children and primary and secondary school students was 50.2% in 2019.^13^ Our data showed that the overall prevalence of myopia among children and adolescents aged 6–16 years was 54.71% in 2021. Ministry of Education of the People’s Republic of China said that the rate of myopia among Chinese primary and secondary school students increased by 11.7% in the first 6 months in 2020.^14^ Reduced outdoor activities, increased time on electronic screens and near work may be cause the increase of prevalence and incidence of myopia in children and adolescents.^15 16^ A million scale observational studies in Wenzhou, Zhejiang province, China, reported that the rate of myopia was 59.35% in students.^17^ According to a city-wide study from Weifang, Shandong province, China, the myopia prevalence was 75.35% among elementary, middle and high schools’ students in 2020.^5^ The reason for difference may be related to underlying factors, such as lifestyle, academic stress, age range or genetic factors.

Agreeing with substantial existing literature, this study found that the prevalence of myopia increased gradually by age. Mountjoy *et al* suggested that exposure to more years of education contributes to the rising prevalence of myopia.^18^ Zhang *et al* found that every additional year at school was associated with a decrease in mean SE refractive error of −0.17 D/y.^19^ Myopia prevalence increased with age, which may be attributed to the lengthy learning period and heavier academic stress in students of older age in China. The higher the age, the heavier the extracurricular homework and the longer the eye use.

Wang’s cross-sectional study concluded that the progression of myopia started to increase dramatically in grade 2 and the change slowed down in grade 8,^20^ which is congruent with our research, demonstrating that the most dramatic increase in prevalence was at 8 years (grade 2) and the increasing slowed down at 14 years (grade 8). More importantly, this trend remained the same even when we adjusted regional and sex differences by calculating the region-standardised prevalence and sex stratification. These findings strongly suggested that myopia tended to develop and progress fastest at 8 years and the stage of stabilisation was at 14 years and afterward. Children start primary school from the age of 6–7 in the Chinese education system. Once children enter school, there is generally increased near-work time, leading to the broken balance between axial elongation and loss of lens power. Myopia developed rapidly with myopic shifts in refraction during this period. After the age of 14, the progress of myopia is slow because of the slow physiological growth in axial length during puberty.^21^ Therefore, myopia control efforts should focus on primary school students. Simultaneously, the prevalence of high myopia was particularly prominent in the older age groups and that in the age of 16 years was up to 16.25%. Therefore, this suggested that we cannot ignore the prevention and control of high myopia for the older age groups to avoid causing pathological complications.^22^

This study suggested that children and adolescents living in the six central districts had the highest prevalence of moderate myopia and high myopia. But our results are incongruent with one existing evidence that indicated the severity of myopia in urban–rural fringes and rural areas.^5^ Students live in central districts in Tianjin with the best educational resources and the greatest study burden. Schools were closed during the COVID-19 pandemic, but students learning continued online. Students who live in central districts may have more time near digital screen and less time for outdoor activities. These factors may negatively impact the onset and development of myopia among school-aged children.^11 23^ Thus, the prevention of myopia in six central districts is worthy of attention.

The strength of current study is a large sample size and city-wide scale research. Moreover, the regions-standardised prevalence and the chain growth rate of myopia were calculated to describe the development and progression of myopia. However, there are limitations to this study. First, this study’s data, collected between March and June in 2021, do not totally represent the changes of myopia prevalence during the 3 year pandemic. Second, non-cycloplegia refraction may overstate the prevalence of myopia in children since cycloplegia refraction was not used in this investigation due to the large sample size (n=864 828). However, when UCVA is combined with non-cycloplegia refraction in serial order, a higher sensitivity is achieved than with either of the two tests alone. The sensitivity of UCVA, non-cycloplegia refraction, and the combination test were 63.55%, 78.50% and 84.35%, respectively.^21^ The combination test was performed in this study. Thus, the present results were relatively reliable. TCARE was carried out annually, and UCVA and non-cycloplegia refraction were tested due to a large number of affected populations. Future studies with cycloplegic refraction in the small sample size are planned to evaluate other potential factors. Finally, the study population was all from Tianjin, which is one of the four municipalities in northern China with higher economic level. The representativeness of the present results in China is limited.

## Conclusions

Our data demonstrated that the burden of myopia in Tianjin is substantial during COVID-19 pandemic. The overall prevalence of myopia was 54.71% in children and adolescents aged 6–16 years. Children and adolescents living in the six central districts had the highest prevalence of moderate myopia and high myopia, and girls had a higher prevalence than boys. The greatest prevalence of myopia shifts occurred at 8 years, with slowing down at 14 years. For policy-makers, intervention in the lower age group may be important to control myopia progression.

## Data Availability

No data are available.
