# Supplementary material for: Prevalence of myopia among children and adolescents aged 6–16 during COVID-19 pandemic: a large-scale cross-sectional study in Tianjin, China
Source: Br J Ophthalmol. 2023 Jul 6;108(6):879–83. doi: 10.1136/bjo-2023-323688 (PMC11137472; doi:10.1136/bjo-2023-323688)
Supplement: Supplementary data [file bjo-2023-323688supp001.pdf]

eTable 1 Prevalence of severity of myopia in different ages [%(95%*CI*)]

Age, y	Total	Myopia		
		Mild	Moderate	High
6	19.04(18.96-19.12)	16.91(15.81-18.01)	1.88(1.48-2.28)	0.25(0.10-0.39)
7	18.78(18.70-18.86)	16.81(16.58-17.04)	1.77(1.68-1.85)	0.20(0.17-0.23)
8	28.01(27.92-28.11)	23.94(23.68-24.20)	3.77(3.65-3.88)	0.31(0.27-0.34)
9	38.82(38.72-38.92)	30.77(30.49-31.05)	7.42(7.26-7.58)	0.63(0.58-0.68)
10	50.52(50.42-50.63)	36.58(36.28-36.89)	12.66(12.45-12.88)	1.28(1.20-1.35)
11	59.90(59.80-60.00)	38.99(38.67-39.30)	18.40(18.15-18.65)	2.52(2.42-2.62)
12	68.27(68.17-68.37)	39.56(39.24-39.89)	24.44(24.15-24.72)	4.27(4.13-4.40)
13	74.34(74.25-74.44)	37.62(37.30-37.95)	29.92(29.61-30.22)	6.80(6.64-6.97)
14	78.46(78.37-78.54)	34.74(34.40-35.08)	34.33(33.99-34.67)	9.39(9.18-9.59)
15	80.91(80.82-80.99)	32.25(31.89-32.61)	36.70(36.33-37.07)	11.96(11.71-12.21)
16	84.81(84.74-84.89)	28.46(28.08-28.83)	40.11(39.70-40.52)	16.25(15.94-16.56)

Total myopia: SE < -0.50D; mild myopia: -3.00D ≤ SE < -0.50D; moderate myopia: -6.00D ≤ SE < -3.00D; high myopia: SE <-6.00D.
